# Neural compensation in persons with HIV and marijuana use: Insights from a reorganized DMN

**DOI:** 10.1162/NETN.a.513

**Published:** 2026-01-28

**Authors:** Mohsen Bahrami, Paul J. Laurienti, Sheri L. Towe, Ryan P. Bell, Heather M. Shappell, Christina S. Meade

**Affiliations:** Laboratory for Complex Brain Networks, Wake Forest School of Medicine, Winston-Salem, NC, USA; Department of Radiology, Wake Forest School of Medicine, Winston-Salem, NC, USA; Department of Translational Neuroscience, Wake Forest School of Medicine, Winston-Salem, NC, USA; Department of Biostatistics and Data Science, Wake Forest University, Winston-Salem, NC, USA

**Keywords:** HIV, Marijuana, fMRI, Brain networks, DMN

## Abstract

The interactive effects of HIV and marijuana (MJ) on the brain remain largely unknown, despite the prevalence of cognitive implications in this population. This study examined the impacts of HIV and MJ on brain networks crucial for normal cognitive function. Functional MRI data and a battery of neuropsychological tests from 237 HIV+ and HIV− adults aged 25–59 years, stratified by MJ use, were collected. The following hypotheses were then tested: (a) HIV is associated with widespread disruption of the small-world organization of the default mode network (DMN) that is exacerbated by MJ use; (b) observed differences are reflected in cognitive performance. Clustering coefficient and global efficiency were used to measure small-world organizations. We found significant differences in the DMN’s clustering and efficiency between our control group (HIV−MJ−) and the other three groups (HIV+MJ−, HIV−MJ+, and HIV+MJ+). In those with HIV/MJ, the DMN reorganized toward a network explained by efficiency than clustering. Global cognitive performance was associated with this group difference. Unlike the control group, participants with HIV/MJ showed an integrated DMN across all cognitive scores. The higher integrity of the DMN in patients with HIV and MJ use (particularly when co-occurring) across cognitive scores could imply compensation to preserve cognitive function.

## Author Summary

Our results indicate that the functional organization of the default mode network, which is a crucial network for normal cognitive performance, is significantly altered by HIV, particularly when co-occurring with marijuana use. However, this change suggests compensation in the brain to maintain normal cognitive function, at least initially, in our relatively young cohort.

## INTRODUCTION

Many people living with human immunodeficiency virus (HIV) develop central nervous system complications, including impairments in motor and cognitive function. While the introduction of more efficient antiretroviral therapies has substantially reduced the prevalence and severity of HIV-associated neurocognitive disorders (HAND), up to 50% of people with HIV (PWH) still experience mild forms of neurocognitive impairment (Heaton et al., 2010; Wang et al., 2020). Moreover, with now near-normal lifespans, many PWH are aging, raising further concerns about the cognitive consequences of HIV, given the known impacts of aging on cognitive reserve (Cody & Vance, 2016). Nevertheless, the neural mechanisms that underlie cognitive deterioration or resilience in this population are poorly understood (Ventura et al., 2018). Elucidating the neural mechanisms will be a critical step toward developing a more accurate approach to identification and classification of neurocognitive impairment in PWH (Elendu et al., 2023; Harezlak et al., 2011; Nightingale et al., 2023). Notably, the milder and more heterogenous presentations of HAND in the modern era of combination antiretroviral therapies have elevated the challenge of early diagnosis and management of neurologic complications (Hakkers et al., 2017), underscoring the importance of research on more sensitive markers that leverage mechanistic impacts of infectious diseases on the brain.

Over the past 2 decades, an increasing number of studies have shown the promise of functional magnetic resonance imaging (fMRI) to uncover the mechanistic impacts of HIV on brain activity (Chang et al., 2001; Chen et al., 2023) and connectivity (Lew et al., 2023; Thomas et al., 2013). These studies have generally found that chronic HIV is associated with altered functioning in subnetworks critical to efficient cognitive functions. Task-based fMRI studies generally find HIV-related hyperactivity in subnetworks implicated in executive control, including prefrontal, posterior parietal, and cingulate cortices, possibly as a compensatory mechanism for maintaining cognitive performance in the face of neural injury (Castelo et al., 2006; Chang et al., 2004, 2008; Connolly et al., 2014; Ernst et al., 2009; Maki et al., 2009; Meade et al., 2016; Melrose et al., 2008). Resting-state studies have found that HIV is associated with diminished functional connectivity both within and between subnetworks that are important for successful cognition, including default mode, executive control, and salience networks (Ann et al., 2016; Ipser et al., 2015; Thomas et al., 2013; Wang et al., 2011). However, prior studies have not adequately examined how HIV may disrupt neural organization that underlies effective cognitive performance.

Cognitive function is supported by networks that likely exhibit many-to-many mapping, with a given cognitive process being supported by multiple networks and vice versa (Pessoa, 2014). Thus, it is essential to study the topology of functional brain networks at global and local levels. Given this complexity, the shift toward milder forms of HAND highlights the need to examine topology/organization of brain networks to better understand the neural mechanisms underlying the subtle and multifaceted effects of HIV on cognitive performance. A potential factor that may contribute to differential effects of HIV on cognitive/brain function is substance use. Marijuana (MJ) use is disproportionately common among PWH, with a reported past-year prevalence of 34% compared with 11% for people without HIV (Montgomery et al., 2019). MJ use has independently been associated with varying degrees of cognitive impairment (Dellazizzo et al., 2022), although some research also suggests it might have neuroprotective effects in PWH (Watson et al., 2020). Additional research is needed to clarify contradictory findings (Towe et al., 2020).

Network neuroscience methods based on graph theory, which have become the cornerstone of characterizing complex systemic impacts of disease on brain networks (Bullmore & Sporns, 2009), provide excellent tools to examine how HIV impacts brain networks across individuals with varying levels of MJ use. There are several network characteristics that are of particular interest as they relate to how the brain processes information. Segregation in a network is achieved by dense clustering among related nodes resulting in specialized neural processing. Integration is achieved through short path length among such clusters and supports distributed neural processing. A network that exhibits both integration and segregation is said to exhibit small-world properties, which have been recognized as a key architectural feature of a cognitively normal brain (Bassett & Bullmore, 2006). Numerous studies have shown that changes in this brain organization can manifest as cognitive dysfunction (Dai & He, 2014; Kambeitz et al., 2016; Liao et al., 2017). In this study, we primarily focused on the small-world organization of the default mode network (DMN), as the key local subnetwork in our analyses. It has been demonstrated that HIV affects DMN connectivity (Flannery et al., 2022; Thomas et al., 2013) and the DMN plays a critical role across a diverse set of cognitive processes (Shen, 2015; Smallwood et al., 2021). However, to our knowledge, no study has examined the impacts of co-occurring HIV disease and MJ use on the DMN organization, and how any potential impact on the DMN organization relates to cognitive performance.

Using network neuroscience methods and leveraging resting-state fMRI and global cognitive performance data from a relatively large cohort of adults stratified by HIV status and MJ use, we aimed to address the following questions: (a) if/how HIV, independently and interacting with MJ, impacts functional organization of brain networks at local and global levels; (b) if/how any HIV/MJ-related brain network characteristics are associated with cognitive performance. More specifically, we tested the hypothesis that HIV would be associated with widespread disruption of the small-world organization of functional brain networks and that MJ use would exacerbate the HIV-related impacts on the brain. We also tested the hypothesis that the level of network disruption associated with HIV and MJ use would be reflected in cognitive performance.

## RESULTS

### Study Cohort

A total of 249 participants were scanned to collect fMRI and high-resolution structural MRI data. After quality control assessments and preprocessing, 12 participants were removed due to excessive head motion, inability to complete MRI, or ineligibility after the scan. The final data used in this study involved 237 participants. Table 1 shows demographic characteristics for each study group. We controlled for age, sex, education, race, and head motion in our statistical models.

**Table 1.** Study cohort demographics

	HIV−MJ− (*n* = 59)	HIV+MJ− (*n* = 62)	HIV−MJ+ (*n* = 53)	HIV+/MJ+ (*n* = 63)
Age	38.86 ± 9.23	39.09 ± 8.09	36.43 ± 7.83	37.81 ± 8.68
Sex	44/15	51/11	41/12	53/10
Education	16.24 ± 2.13	15.35 ± 2.60*	14.33 ± 2.38*	14.22 ± 2.41*
Race	35/24	38/24	36/17	45/18
Average RMD	0.10 ± 0.03	0.10 ± 0.05	0.10 ± 0.04	0.11 ± 0.05
GlobalT	50.45 ± 5.91	48.85 ± 5.29	50.59 ± 5.65	47.27 ± 5.21*

GlobalT, Education, and Average_RMN: mean ± std; Sex: male/female; Race: Non−White/White.

* Significant difference with the reference group (*p* value < 0.05) according to a two-tailed *t*-test analysis. *p* values for comparing all groups are shown in Supporting Information Table S11. Despite significant differences in GlobalT scores, all four groups fall within the normal cognitive range.

### Group Differences in the DMN Organization

Key results from our primary analyses (Model 1) that compared groups on clustering and efficiency of the DMN and all other brain regions (as a whole) are presented in Table 2.

**Table 2.** Key results from primary analysis

Connection strength − clustering			Connection strength − efficiency		
Parameter	Estimate	*p* value*	Parameter	Estimate	*p* value*
HIV+MJ− / Within DMN	−0.0015	0.7862	HIV+MJ− / Within DMN	0.0120	**0.0049**
HIV−MJ+ / Within DMN	−0.0116	**0.0466**	HIV−MJ+ / Within DMN	0.0216	**<0.0001**
HIV+MJ+ / Within DMN	−0.0179	**0.0061**	HIV+MJ+ / Within DMN	0.0367	**<0.0001**
HIV+MJ− / Outside DMN	0.0036	0.4643	HIV+MJ− / Outside DMN	−0.0026	0.4487
HIV−MJ+ / Outside DMN	0.0037	0.4708	HIV−MJ+ / Outside DMN	−0.0024	0.5023
HIV+MJ+ / Outside DMN	0.0025	0.6103	HIV+MJ+ / Outside DMN	−0.0019	0.5679

* Adjusted using the adaptive False Discovery Rate (FDR) correction procedure. **Bold** values show significant results. Estimates and *p* values show the mean difference with respect to the reference group (HIV−MJ−).

**Figure 1.**
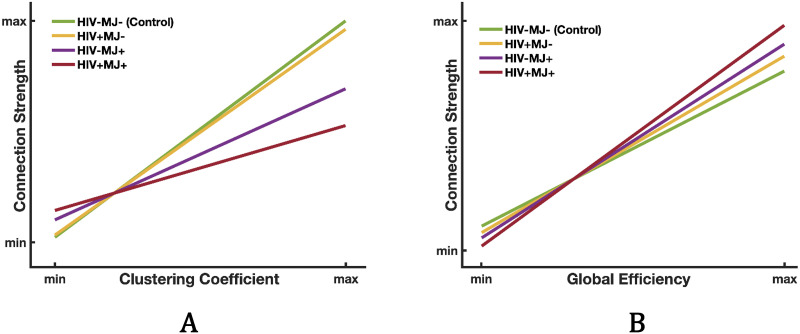
Line plots demonstrating the relationship of the DMN connection strength with clustering (A) and efficiency (B) for all groups. Group differences identified through the statistical analysis (Table 2) are the differences in the *slopes* in the line plots. For clustering (A), the control group (green) has the steepest slope, showing stronger connections within the DMN as clustering increases. The slope becomes slightly less positively steep for HIV+MJ− (yellow) and significantly less positively steep for HIV−MJ+ (purple) and HIV+MJ+ (red). For efficiency (B), a reverse pattern is observed, where HIV+MJ+ has the steepest slope, showing the strongest connections among the DMN’s nodes with higher global efficiency. Confidence intervals for each relationship are shown in Supporting Information Figure S2.

Parameter estimates and corrected *p* values quantify the group difference between the HIV−/MJ− group (the controls) and the other three groups. Within the DMN, the two MJ+ groups (HIV+/MJ+ and HIV−/MJ+) differed from the control group for clustering, and all three groups (HIV+/MJ−, HIV−/MJ+, and HIV+/MJ+) differed from the control group for efficiency. There were no group differences in these metrics outside of the DMN. To better understand these group differences, the results are depicted graphically in Figure 1. Overall, the DMN nodes have increased connection strength as both clustering and efficiency increase, but the slopes of the line plots differ across the groups. Figure 1A shows that the control group has a steeper slope compared with HIV−/MJ+ and HIV+/MJ+. This suggests that, for people who use MJ, the DMN nodes are less dependent on their clustering, particularly with co-occurring HIV. Figure 1B shows a reverse pattern, wherein the HIV+MJ+ group had the steepest slope relative to the control group, suggesting that the DMN nodes have stronger connection as their efficiency increases. The HIV−/MJ+ and HIV+/MJ− also have steeper slopes relative to the control group.

The slope differences between each of the groups and the HIV−/MJ− are significant, as shown in Table 2. Confidence intervals for each relationship shown in Figure 1 is shown in Supporting Information Figure S2.

### Group Differences in Association of the DMN Organization and GlobalT

Table 3 presents key results from our secondary analysis (Model 2) that examined the group differences in the association of the clustering/efficiency and GlobalT within and outside of the DMN. Parameter estimates and corrected *p* values in this table quantify the group difference between the HIV−/MJ− group (the control) and the other three groups. Specifically, this table shows if/how the moderating effects of GlobalT on the relationship of connection strength with clustering/efficiency differs between the control group and the other three groups. As in Model 1, the significant group differences were only within the DMN. For both clustering and efficiency, all three groups were significantly different from the HIV−/MJ− group.

**Table 3.** Key results from secondary analysis

Connection strength − clustering × *GlobalT*			Connection strength − efficiency × *GlobalT*		
Parameter	Estimate	*p* value*	Parameter	Estimate	*p* value*
HIV+MJ− / Within DMN	0.0227	**<0.0001**	HIV+MJ− / Within DMN	−0.0208	**<0.0001**
HIV−MJ+ / Within DMN	0.0174	**0.0019**	HIV−MJ+ / Within DMN	−0.0160	**0.0002**
HIV+MJ+ / Within DMN	0.0207	**0.0003**	HIV+MJ+ / Within DMN	−0.0214	**<0.0001**
HIV+MJ− / Outside DMN	−0.0064	0.2066	HIV+MJ− / Outside DMN	0.0043	0.2304
HIV−MJ+ / Outside DMN	0.0011	0.8254	HIV−MJ+ / Outside DMN	−0.0005	0.8776
HIV+MJ+ / Outside DMN	0.0031	0.5444	HIV+MJ+ / Outside DMN	−0.0030	0.4057

* Adjusted using the adaptive FDR procedure. **Bold** values show significant results. Estimates and *p* values show the difference with respect to the reference group (HIV−MJ−).

**Figure 2.**
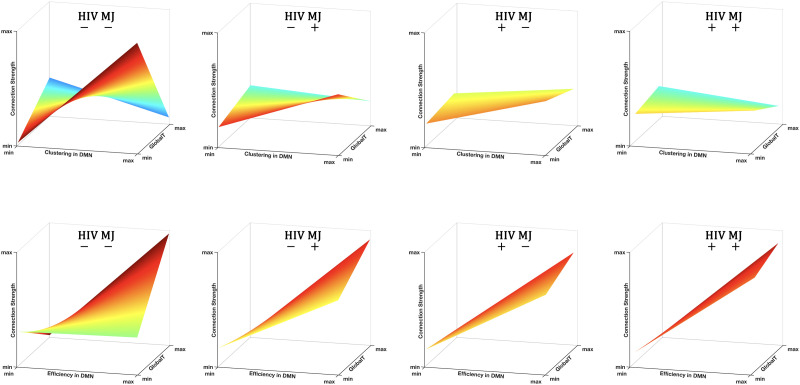
Visualization of the group differences in the association of GlobalT and DMN organization. The surface plots show how, for each group, the relationship between the connection strength and clustering (A) and efficiency (B) in the DMN (i.e., the slope) changes as GlobalT increases. The color shows the slope for each unique GlobalT. As GlobalT increases, the slopes for both clustering (A) and efficiency (B) change significantly for the control (HIV−MJ−) group, but this shift is diminished for the PWH or people with MJ, with the HIV+MJ+ group having the lowest shift in the DMN’s reorganization. Nevertheless, a stronger positive slope for the connection strength − efficiency relationship and a slope close to zero for the connection strength − clustering relationship for smaller GlobalT values could imply compensation in the DMN (as detailed in the Discussion section).

The estimates of the GlobalT effects were larger in all groups compared with the control group (positive estimates) for clustering but were smaller for efficiency (negative estimates). The significant findings in Table 3 are illustrated by surface plots in Figure 2. The surfaces are colored by the slope of the connection strength − clustering relationship (Figure 2A) and the connection strength − efficiency relationship (Figure 2B) across GlobalT values. In the control group (HIV−/MJ−), at higher GlobalT scores, the slope for clustering was lower while the slope for efficiency was higher. This indicates that there was a significant difference in DMN connectivity at different levels of GlobalT. Specifically, high efficiency nodes within the DMN have stronger connections with each other in individuals with higher GlobalT. However, the connection strength among DMN nodes with higher clustering is weaker in those with higher GlobalT. This transition from higher dependence on clustering at lower GlobalT to higher dependence on efficiency at higher GlobalT in the control group may imply a shift in the organization of the DMN, where the DMN is more integrated in order to more efficiently process neural information in individuals with higher GlobalT.

**Figure 3.**
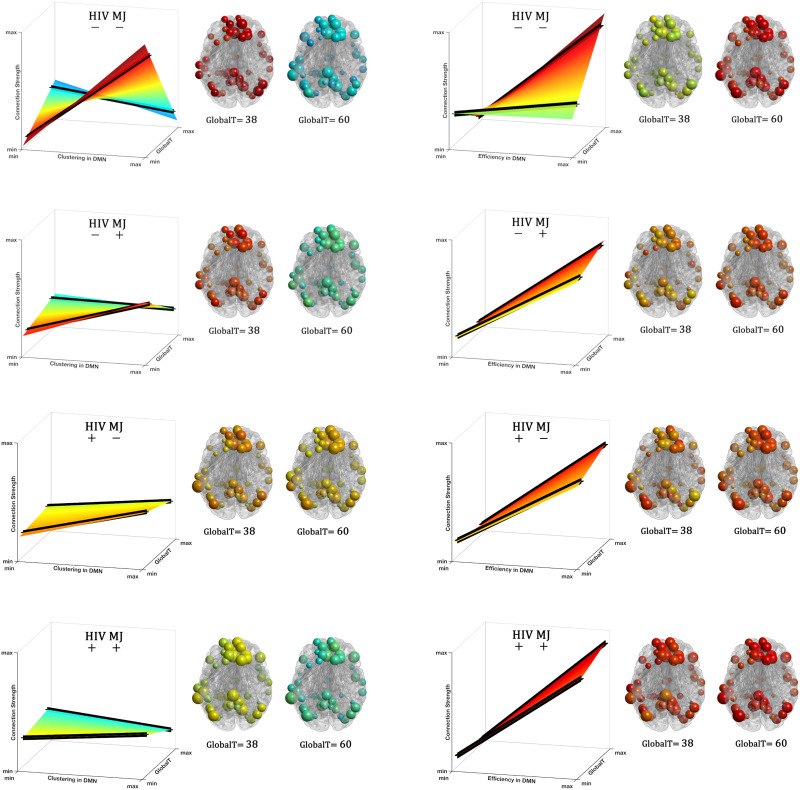
Visualization of the implied DMN organization for the same GlobalT across different groups. Solid lines on each surface plot show the relationship between the connection strength − clustering (A) and strength − efficiency (B) for two unique GlobalT values (38 and 60). Group representative networks are shown for individuals with low (38) and high (60) GlobalT for each group. As this figure shows, with respect to both clustering and efficiency, brain networks of people with lower GlobalT and with HIV, MJ, or particularly both, resemble networks with higher GlobalT in the control group. This could imply compensation in the DMN as detailed in the Discussion section. Nodes in these networks are colored by the slopes and sized by their actual clustering (A) and efficiency (B). Please see the Supporting Information for more details about generating the group representative networks from the obtained slopes for each group.

This substantial shift in the DMN’s reorganization related to GlobalT in the control group is diminished in individuals with HIV and/or MJ. As shown in Figure 2, these groups demonstrate substantially smaller shifts in the surface plots at increasing GlobalT scores, with HIV+MJ+ group showing the least shift in the relationship between the DMN organization and GlobalT. The DMN organization for PWH and/or people with MJ is predominantly associated with nodal efficiency (depicted as the overall steep slope in Figure 2B), a finding that is recapitulated in Figure 1. There was very weak dependence on nodal clustering (depicted as an almost flat surface in Figure 2A) even for individuals with low GlobalT, also consistent with Figure 1. Figure 3 illustrates the slopes at low and high GlobalT (***low: 38, high: 60***), shown as two solid lines on the surface plots, across the four groups. To aid in interpreting the slope differences between individuals with high and low GlobalT, representative group networks constructed for each level are shown. Nodes in these networks are colored by the slopes and sized by their actual network metrics (clustering in Figure 3A and efficiency in Figure 3B). As Figure 3 shows, there was a substantial difference in the topology of the network between high and low GlobalT for the control group (HIV−/MJ−). This difference between high and low GlobalT was substantially reduced in the other groups.

## DISCUSSION

Network science approaches allow us to better understand how neurological conditions may disrupt neural organization, specifically the interaction between integration and segregation, that underlies cognitive function. The overarching goal of this study was to test the hypothesis that HIV, particularly when combined with chronic MJ use, would be associated with significant alterations in the small-world organization of the DMN that are manifested in cognitive performance. To examine potential detrimental or compensatory mechanisms in the brain, we used a novel statistical model to assess the DMN organization (specifically, clustering and efficiency, two hallmark metrics of a small-world organization) within its global context and thus accounting for its interactions with other brain regions. This was done while also controlling for potential confounding effects such as age and education (Bahrami et al., 2019a). The utilized statistical framework was specifically developed for testing hypotheses on brain networks. This framework allowed for examining complex system-level changes in the brain and disentangling compensatory mechanisms. Identifying such complex changes is particularly important when examining younger populations (as in the current study) as neural compensation may go undetected by simpler models like standard *t*-test analyses. Our previous studies have also demonstrated the strong potential of our statistical methodology in identifying complex compensatory changes (Bahrami et al., 2023). This makes the reported results here more reliable, robust, and independent of those obtained from simpler models, due to their inherent limitations (Bahrami et al., 2022). To the best of our knowledge, this study used the largest sample size to date to explore both independent and interactive effects of HIV and MJ on functional organization of the brain at DMN (local) and whole-brain (global) levels simultaneously.

Our results show the significant impact of HIV and MJ on the clustering and efficiency within the DMN. Our primary model showed a difference in the DMN organization in individuals with HIV and/or MJ, and those with co-occurring HIV and MJ showed the greatest difference. The observed difference was only significant in the DMN (i.e., not statistically significant in non-DMN brain regions). When compared with the control group, those with HIV/MJ showed significantly weaker connections between nodes with higher clustering but significantly stronger connections between nodes with higher efficiency in the DMN, suggesting a DMN reorganization toward a network that is more associated with efficiency than clustering. A similar study focused on HIV (Ventura et al., 2018) showed that the efficiency of the posterior cingulate cortex (PCC), a key region in the DMN, is significantly higher in PWH compared with the control group. Our results replicate and expand on this finding. We believe that the predominant reliance on efficiency suggests a gradual but significant increase in the DMN integrity (i.e., stronger connections among nodes with higher efficiency in distributing the neural information), with additive effects from MJ such that the HIV+MJ+ group had the greatest difference from the control group. Due to the importance of network integrity (Weiler et al., 2018), particularly the DMN’s integrity (Dennis & Thompson, 2014), in maintaining normal cognitive function, we believe that our results could indicate compensation in individuals with HIV and/or MJ. This is again consistent with the conclusion in Ventura et al. (2018) that links the higher PCC efficiency to compensation.

Our secondary model examined the relationship between cognitive performance and the DMN organization. In the control group, the DMN organization differed substantially when comparing persons with low cognitive performance (highly associated with clustering) to those with high cognitive performance (highly associated with efficiency). This substantial difference suggests a shift from a more distributed network to a more integrated network as cognitive performance improves. This difference in the DMN organization as a function of cognitive function is largely diminished for the other three groups, with those with both HIV and MJ having the least difference in the DMN organization between high and low cognitive performers. The DMN organization in these three groups suggests that maintaining cognitive performance required compensatory shifts in the DMN’s topology as their DMNs were predominantly associated with efficiency over clustering (a more integrated DMN) across almost all GlobalT values. In other words, for participants with HIV and/or MJ, the DMN tends to be more integrated even for lower levels of cognitive performance; to maintain cognitive performance in the face of HIV and MJ use, the DMN has already reorganized toward a more integrated topology, which is aligned with our primary results. This would suggest compensation in the brain in individuals with HIV/MJ.

Altogether, our findings support a reorganized DMN in PWH, particularly in the context of chronic MJ use, to maintain functional abilities. The independent and interactive effects of HIV and MJ on functional brain networks have been studied in an increasing number of studies. Using rs-fMRI data, Minosse et al. (2021) showed a similar change in the whole-brain global efficiency, where PWH had significantly higher global efficiency when compared with healthy subjects. A study by Hall et al. (2021), which used similar groups to our study, showed a significant change in several network metrics including global efficiency for multiple network densities. However, the reported differences, including higher global efficiency, was limited to HIV only (HIV+/MJ−) and MJ only (HIV−/MJ+) groups, while the HIV+/MJ+ group was not different from the control group on whole-brain metrics. The authors hypothesized that this is due to MJ normalizing the impact of HIV. Although several other studies have raised the same notion about MJ modulating the impacts of HIV (Christopher-Hayes et al., 2021; Schantell et al., 2022; Thames et al., 2017; Watson et al., 2020), a systematic review presents some contradictory evidence in which MJ use has been linked to worsening the compensatory mechanisms in the brain (Skalski et al., 2016). Our study, which had a sample size three times larger than that in Hall et al. (2021) and focused on the DMN rather than whole-brain metrics, provides novel insight into how MJ use may indeed contribute to cognitive function. Our results indicate that in PWH who also use MJ, the DMN, at least initially, reorganizes more substantially to preserve functional abilities. It is important to note that our sample is overall quite young, and thus the reorganization found in this study may not yet have overt clinical implications but precede more observable clinical implications.

This cross-sectional study is not without limitations. While our results shed some light on current controversies regarding MJ effects in the literature, the main outcomes showing group differences, and their associations with cognitive performance cannot establish causality. Thus, future longitudinal studies are clearly warranted to elucidate the ultimate neurological impacts of HIV and MJ. Our analysis was focused on the DMN at the local level and a global network comprising all other brain regions combined. Due to the sophisticated statistical model and the large number of interaction covariates that we employed for testing our hypotheses, breaking the global network into more specific networks (i.e., examining multiple brain subnetworks) was not feasible. However, we acknowledge that combining all other brain regions into one big network could mask important group differences in specific networks such as the frontotemporal, dorsal attention, and salience networks. Future studies that target more specific subnetworks outside the DMN may reveal additional significant changes. We focused on the DMN as its reorganization, including alterations in its clustering and efficiency, has been a major focus of numerous studies investigating cognitive functions and the neurological basis of various brain disorders (Hafkemeijer et al., 2012; Mohan et al., 2016; Smallwood et al., 2021) over the past 2 decades. However, exploratory analyses of other networks linked to HIV and MJ are warranted. It should also be noted that although we did not explicitly test within versus between network effects (i.e., DMN vs. other brain regions), our analyses examined the DMN within the context of the whole brain, thereby accounting for its interactions with other brain regions. Altered connectivity between the DMN and other brain regions could be an underlying cause of the altered efficiency/clustering within the DMN. Lastly, our analysis is performed on one static (aggregate) network estimated for each participant using the entire length of their scan. In contrast, dynamic brain network analyses have been introduced and have proven to provide profound insight into behavioral shifts and adaptive processes in normal and abnormal brain function. Dynamic network analyses produce a series of networks spanning the duration of an individual scan. Our future work will also focus on performing a dynamic connectivity analysis of these data to investigate how individuals are transitioning in and out of different network topologies and whether these dynamic properties are related to HIV and MJ.

In summary, our results show that in individuals with HIV and/or MJ, the DMN reorganizes toward a network that is predominantly associated with efficiency than clustering, and global cognitive performance is associated with this reorganization. At increasing levels of cognitive performance in the control group, the small-world organization of DMN shifts from a more distributed network to a more integrated network. In contrast, this shift substantially diminishes in participants with HIV and/or MJ use (particularly for the HIV+MJ+ group), where their DMN shows an integrated network across almost all cognitive scores. This could imply compensation to preserve cognitive function in our relatively young cohort. This study also lays the groundwork for future studies to explore other subnetworks, such as frontotemporal subnetwork.

## MATERIALS AND METHODS

### Participants and Procedures

This study was open to HIV+ and HIV− adults aged 25–59 years who were stratified by MJ use status. Participants were recruited from the Raleigh-Durham area in North Carolina, USA, using advertisements and flyers in local community-based organizations, websites, and medical clinics. Individuals who met preliminary eligibility criteria were invited for an in-person screening visit. After providing written informed consent, participants completed questionnaires and clinical interviews on substance use as well as medical and psychiatric history, urine drug testing, urine pregnancy testing (for participants of child-bearing potential), and HIV screening. For participants with known HIV disease, HIV+ status was verified via medical record review. For all others, a rapid HIV−1/2 antibody test was conducted using a droplet of whole blood collected by pinprick; all participants who were screened had a nonreactive test result. PWH were required to be in HIV care, on a stable antiretroviral therapy regimen for >1 year, and had plasma HIV RNA <200 copies/ml at the last clinic visit (and confirmed at the study visit). The MJ+ group met the following criteria: ≥4 days of cannabis use in the past 30 days or a THC-positive urine drug screen; ≥1 year of regular lifetime cannabis use; and smoking as the primary route of cannabis administration. MJ− was defined as 0 days of cannabis use in the past 30 days, a THC-negative urine drug screen, and no history of regular cannabis use. In both groups, alcohol and nicotine use were permitted.

Exclusion criteria were nonfluency in English, illiteracy, severe head trauma with loss of consciousness >30 min and persistent functional decline, neurological disorders or serious neurological events without return to normal cognition, severe mental illness, and MRI contraindications, including pregnancy. For substances other than MJ, participants were excluded for a positive urine drug screen, ≥2 days of use in the past 30 days, any regular use in the past 5 years, or >2 years of regular lifetime use. The urine drug toxicology test assessed the presence of 11 substances (MJ, cocaine, opiates, methamphetamine, amphetamine, benzodiazepines, barbiturates, methadone, buprenorphine, ecstasy, and oxycodone). Substance use and related problems were assessed using the Addiction Severity Index-Lite (McLellan et al., 1992). Duration, frequency, and quantity of lifetime MJ use were assessed using the Lifetime Drug Use Questionnaire (Czermak et al., 2005). Module E of the Structured Clinical Interview for DSM-5 (SCID-5) assessed substance use disorders (First et al., 2015). Modules A and B of SCID-5 assessed lifetime psychiatric disorders (First et al., 2015). Literacy was assessed using the word reading test of the Wide Range Achievement Test 4 (Wilkinson & Robertson, 2006). Medical records were reviewed to ensure no exclusionary medical conditions.

A battery of tests assessed seven domains of neuropsychological function.

1. Executive functioning: Delis-Kaplan Executive Function System (D-KEFS) Tower Test − total achievement score (Delis et al., 2001); Wisconsin Card Sorting Test − total errors (Kongs et al., 2000); Stroop Color and Word Test Interference − difference between actual and predicted score on the Color-Word trial (Golden, 1978); Trail Making Test Part B − number of seconds to completion (Heaton et al., 1991; Reitan & Wolfson, 1993).
2. Working memory: Paced Auditory Serial Addition Task-50 − total number correct (Diehr et al., 2003); Wechsler Adult Intelligence Scale, Fourth Edition (WAIS-IV) Digit Span − total number correct (Wechsler, 2008); WAIS-IV Letter-Number Sequencing − total number correct (Wechsler, 2008); Wechsler Memory Scale, Fourth Edition (WMS-IV) Spatial Addition − total number correct (Wechsler, 2009).
3. Motor ability: Grooved Pegboard Test (dominant and nondominant hands) − number of seconds to completion (Heaton et al., 1991; Reitan & Wolfson, 1993); Finger Tapping (dominant and nondominant hands) − number of seconds to completion (Heaton et al., 1991; Reitan & Wolfson, 1993).
4. Learning (immediate recall): Hopkins Verbal Learning Test–Revised (HVLT-R) − total number of words recalled on trials 1–3 (Brandt & Benedict, 2001); Brief Visual Memory Test–Revised (BVMT-R) − total score for figures recalled on trials 1–3 (Benedict et al., 1996); WMS-IV Logical Memory − total number of story details recalled in Trial I (Wechsler, 2009).
5. Memory (delayed recall): HVLT-R − total number of words recalled on trial 4 (Brandt & Benedict, 2001); BVMT-R − total score for figures recalled on trial 4 (Benedict et al., 1996); WMS-IV Logical Memory − total number of story details recalled in Trial II (Wechsler, 2009).
6. Processing speed: Trail Making Test Part A – number of seconds to completion (Reitan & Wolfson, 1993); WAIS-IV Coding − total number correct items (Wechsler, 2008); Stroop Color and Word Test color naming score − total number of items completed (Golden, 1978).
7. Fluency: Controlled Oral Word Association Test letter and category fluency (letters F, A, and S and animals) − total number of words generated and total number of animals generated (Benton et al., 1983; Heaton et al., 1991); D-KEFS Design Fluency − total unique designs across conditions 1–3 (Delis et al., 2001).

For each test, the most updated scoring software available from the test publisher was used to convert raw scores to demographically adjusted T scores. We computed domain T scores by taking an average of the T scores for all the tests within each domain, and the seven domain scores were averaged to compute a global T score (GlobalT) to estimate overall cognitive performance.

### MRI Acquisition and Preprocessing

#### Image acquisition.

Data were acquired on a GE SIGNA Premier 3 T wide-bore MRI scanner with a 48-channel head coil. High-resolution T1-weighted images were obtained using a BRAVO (BRAin VOlume) sequence with a SENSE (Sensitivity Encoding) factor of 2 (repetition time [TR] = 2,234.94 ms, echo time [TE] = 3.076 ms, inversion time [TI] = 900 ms, voxel size = 0.9375 mm × 0.9375 mm × 1 mm, field of view [FOV] = 240 mm^2^, flip angle = 8°, interleaved slices = 154). Whole-brain blood oxygenation level-dependent (BOLD) images (Ogawa et al., 1990) were collected using T2*-weighted echo-planar imaging with a multiband factor of 3 and an SENSE factor of 2 (TR = 1,500 ms, TE = 25 ms, flip angle = 52°, voxel size = 2.0 mm^3^, FOV = 256 mm^2^, 128 × 128 matrix, 69 interleaved slices). Participants were asked to fixate on a crosshair with their eyes open. Two runs were completed, each with 240 time points. The first six volumes of each run were excluded to ensure that a steady state had been reached. Noise from respiration and cardiac signals were then removed using *RETROICOR* (Glover et al., 2000).

#### Preprocessing and functional network generation.

Functional and structural MRI scans were preprocessed using a standardized pipeline performed using *fMRIPrep* 20.1.0 (Esteban et al., 2019, 2020), which is based on *Nipype* 1.4.2 (Gorgolewski et al., 2011, 2018). This pipeline performs minimal preprocessing, including motion correction, field unwarping, normalization, bias field correction, and brain extraction. Additional details of the fMRIPrep pipeline are provided in the Supporting Information. Postprocessing was performed using *nilearn* (Abraham et al., 2014). The data were detrended and low- and high-pass filtered (cutoffs of 0.08 Hz and 0.008 Hz, respectively). Cerebrospinal and white matter signals were removed along with the six head motion parameters (three rotations and three translations). The data were then standardized across runs before being temporally concatenated for each participant. Only scans with a relative mean displacement (RMD) of <0.3 mm, measured using *FSL*’s *MCFLIRT* with the root mean square intensity difference to the middle volume as the reference, were included; four participants had one run excluded. Using *nilearn* (Abraham et al., 2014), we obtained the time course of brain activity from 300 cortical and subcortical regions (Seitzman et al., 2020) across 234 time points and computed correlations between all pairs of regions, creating a 300 × 300 correlation matrix. Three regions were excluded because at least one participant did not have coverage of at least 50% of voxels in the region. Edges were not calculated if the two regions were <20 mm apart because connectivity between close regions can be artificially inflated due to motion (Power et al., 2012). Connectivity matrices consisted of an undirected weighted matrix for each participant with negative values set to zero.

### Graph Metrics and DMN Mask

Clustering coefficient quantifies the extent to which a node’s neighbors are also connected to each other, forming a triangle. A high clustering coefficient indicates a high level of local connectivity or “cliquishness” in a network. Global efficiency quantifies the overall efficiency of information exchange across the entire brain or a defined subnetwork. Higher global efficiency indicates closely connected nodes with rapid communication or transfer of information. Together, higher clustering coefficient and global efficiency characterize a small-world network, a network that is highly locally clustered while maintaining efficient communication across its clusters.

We used clustering coefficient and global efficiency (a more robust and interpretable alternative to path length; Achard & Bullmore, 2007; Latora & Marchiori, 2001) to quantify small-worldness because they are simple, well-defined, and particularly more interpretable compared with other alternatives that directly quantifying small-worldness using random networks. Moreover, they are often more robust than other alternatives that rely on the model choice for random graphs, which can sometimes yield misleading results about small-worldness (Rubinov & Sporns, 2010). We should also note that network density is critical in examining small-worldness and ensuring accurate findings. To ensure that our results were not driven by network density, we set only the negative correlations to zero as mentioned above. The network density was nearly identical across the four groups with no significant differences between the groups (see Supporting Information Tables S12 and S13 for more details).

We used a brain connectivity toolbox to compute these two network metrics (Rubinov & Sporns, 2010). The mask defining the DMN was created using 65 ROIs from the Seitzman atlas labeled as the DMN. These Regions of Interest (ROIs) were first identified in Power et al. (2011) with additional subcortical and cerebellar ROIs assigned to the DMN using an information-theoretic community detection algorithm (Rosvall & Bergstrom, 2008).

### Statistical Analysis

#### Modeling framework.

We used a multivariate statistical model, developed for testing hypotheses on brain networks (Bahrami et al., 2019a, 2019b), to first identify group differences in brain organization in the DMN and across all other brain regions as a whole (primary analysis − Model 1) and then to investigate if/how any potential group difference would be associated with global cognitive performance assessed using GlobalT (secondary analysis − Model 2). Our statistical models allowed for the analysis of the DMN within its global network context (thus better capturing the compensatory mechanisms in the brain), while controlling for confounding effects including age, gender, education, race, and within-scanner head motion (i.e., average RMD). More specifically, this mixed-effects regression framework provided the quantified relationships (estimates) and statistical inference (*p* value for each estimate) between a set of independent covariates and the strength of brain connections as the dependent covariate. Table 4 summarizes the independent covariates for the primary and secondary analyses.

**Table 4.** Independent variables used in our multivariate statistical regression model

Covariates	Model
Covariates of interest (COIs)	
HIV: Binary variable separating HIV+ and HIV− groups	Models 1 & 2
MJ: Binary variable separating MJ+ and MJ− groups	Models 1 & 2
HIVMJ: Binary variable separating HIV+/MJ+ and HIV−/MJ− groups	Models 1 & 2
GlobalT: Continuous variable quantifying global cognitive performance	Model 2

Network metric covariates (NetMets)	
Clustering coefficient: Nodal clustering coefficient	Models 1 & 2
Global efficiency: Nodal global efficiency	Models 1 & 2

Subnetwork covariate (DMN)	
DMN: Binary variable separating the DMN regions from other brain regions	Models 1 & 2

Interaction covariates	
Interactions of COIs (HIV, MJ, HIVMJ) and NetMets	Models 1
Interactions of COIs (HIV, MJ, HIVMJ) and DMN	Models 1
Interactions of NetMets and DMN	Models 1 & 2
Interaction of GlobalT and other COIs	Model 2
Interactions of COIs (all) and NetMets	Model 2
Interactions of COIs (all) and DMN	Model 2
Interactions of COIs (all), NetMets, and DMN	Model 2
Interactions of GlobalT, other COIs, NetMets, and DMN	Model 2

Confounding covariates	
Dist: Continuous variable for spatial distance between brain regions	Models 1 & 2
Dist2: Square of Dist	Models 1 & 2
Age: Continuous variable representing the age	Models 1 & 2
Sex: Binary variable separating male/female	Models 1 & 2
Race: Binary variable separating White/non-White	Models 1 & 2
Education: Continuous variable for years of education	Models 1 & 2
RMD: Continuous variable for RMD (average head motion)	Models 1 & 2

#### Primary analysis covariates.

As summarized in Table 4, Model 1 included: (1) covariates of interest: grouping variable (HIV+/MJ+, HIV+/MJ−, HIV−/MJ+, and HIV−/MJ−) with HIV−MJ− serving as the control group; (2) network metrics: clustering coefficient and global efficiency; (3) DMN covariate: a binary variable separating DMN regions from other brain regions; (4) interaction effects: all two-way and three-way interactions between covariates in 1, 2, and 3; and (5) confounders: age, education, and so forth. Estimates and *p* values obtained for interaction covariates describe topological differences (i.e., differences in clustering coefficient/global efficiency) in the DMN and other brain regions between groups. To further disentangle the group differences within the DMN and outside of the DMN (all other brain regions), we used post hoc analyses, in which we applied appropriate contrast statements on already estimated residuals obtained from our fitted statistical model. Contrast statements are simply linear combinations of different independent covariates (interaction covariates in our analyses) that test specific hypotheses.

We have provided a detailed description of the contrast statements in the Supporting Information. The contrast statements were used to test if/how the DMN’s topology differed significantly between our control group (HIV−/MJ−) and each of the other groups. In our statistical model, the group difference was measured as the difference in the relationship of connection strength and network metrics (i.e., clustering coefficient and global efficiency). Thus, to better visualize the group differences, we made 2D line plots that showed the relationship between the DMN’s clustering or efficiency (*x*-axis) and the DMN’s connection strength (*y*-axis) across the four groups.

#### Secondary analysis covariates.

Model 2 examined if/how the group differences in the DMN organization would relate to GlobalT. To examine this, we used the same independent covariates from our primary analysis and added GlobalT as well as its interactions with the covariates in 1, 2, and 3. Using contrast statements (see the Supporting Information for more details), we were able to test if/how the relationship between GlobalT and DMN organization differed between the control group and the three other groups. To visualize the results, we made 3D surface plots that showed the relationship between the DMN’s clustering or efficiency (*x*-axis), DMN’s connection strength (*y*-axis), and GlobalT (*z*-axis). The estimates for each unique GlobalT value were obtained using the contrast statements. Finally, and for visualization purposes, we estimated group representative networks in brain space using the statistical model outcomes (see the Supporting Information for more details).

To ensure that the strong correlation between network metrics (used as endogenous covariates in our models) and connectivity (used as dependent variable) did not inflate the standard errors of the interaction terms (resulting in artificially significant relationships), we mean-centered all continuous variables (including network metrics) and found no indications of multicollinearity in our model fits. To further ensure this, we assessed the correlation between network metrics and their corresponding interaction terms (used in our contrast statements) and found no concerning correlations (i.e., none approaching ±1), indicating no problematic collinearity (see Supporting Information Table S14).

## ACKNOWLEDGMENTS

This study was funded by NIH/NIDA: R01DA047149.

## SUPPORTING INFORMATION

Supporting information for this article is available at https://doi.org/10.1162/NETN.a.513.

## AUTHOR CONTRIBUTIONS

Mohsen Bahrami: Conceptualization; Data curation; Formal analysis; Methodology; Writing – original draft; Writing – review & editing. Paul J. Laurienti: Conceptualization; Writing – review & editing. Sheri L. Towe: Conceptualization; Writing – review & editing. Ryan P. Bell: Conceptualization; Data curation; Methodology; Writing – review & editing. Heather M. Shappell: Conceptualization; Writing – review & editing. Christina S. Meade: Conceptualization; Data curation; Funding acquisition; Writing – review & editing.

## FUNDING INFORMATION

NIH/NIDA, Award ID: R01DA047149.

## DATA AVAILABILITY

The software used to analyze the data in this study is publicly available at https://www.nitrc.org/projects/wfu_mmnet, and the additional codes used in estimating the contrast statements are available from the corresponding author, upon request. The Supporting Information file provides a detailed description of making the contrast statements. The data used in this study are not publicly available since they come from different disciplines and contain information that could compromise the privacy of research participants. The data may be provided upon reasonable request.

## Supplementary Material



## TECHNICAL TERMS

DMN: A critical brain subnetwork involved in various cognitive processes and disrupted in different neurological disorders.
fMRI: An imaging technique that uses a sequence of MRI data to measure brain activity.
GlobalT: A cognitive assessment value that aggregates measurements of various cognitive abilities such as attention, memory, and more.
Global efficiency: A metric that quantifies the efficiency of neural information flow in brain networks.
Mixed-effects regression: A regression model that controls for the correlation between repeated measurements from the same subject.
