# Supplementary material for: Neural compensation in persons with HIV and marijuana use: Insights from a reorganized DMN
Source: Netw Neurosci. 2026 Jan 28;10(1):118–36. doi: 10.1162/NETN.a.513 (PMC12956298; doi:10.1162/NETN.a.513)
Supplement: Supplementary file 1 [file netn-10-1-118-s001.pdf]

Supplemental Material

MRI pre-processing

Anatomical data preprocessing. The T1w image was corrected for intensity non-uniformity with N4BiasFieldCorrection [1], distributed with ANTs 2.2.0 [2], and used as T1w-reference throughout the workflow. The T1w-reference was then skull-stripped with a *Nipype* implementation of the antsBrainExtraction.sh workflow, using OASIS30ANTs as the target template. Brain tissue segmentation of cerebrospinal fluid (CSF), white-matter (WM) and gray-matter (GM) was performed on the brain-extracted T1w using FAST [FSL 5.0.9, 3]. Brain surfaces were reconstructed using recon-all [Freesurfer 6.0.1, 4], and the brain mask estimated previously was refined with a custom variation of the method to reconcile ANTs-derived and FreeSurfer-derived segmentations of the cortical GM of Mindboggle [5]. Volume-based spatial normalization to MNI152NLin6Asym standard space was performed through nonlinear registration with antsRegistration [6], using brain-extracted versions of both T1w reference and the T1w template.

Functional data preprocessing. For each BOLD run, the following preprocessing was performed. First, a reference volume and its skull-stripped version were generated. Head-motion parameters with respect to the BOLD reference (transformation matrices, and six corresponding rotation and translation parameters) were estimated before any spatiotemporal filtering using mcflirt [FSL 5.0.9, 7]. BOLD runs were slice-time corrected using 3dTshift from AFNI 20160207 [8]. A deformation field to correct for susceptibility distortions was estimated based on *fMRIPrep*'s *fieldmap-less* approach. The deformation field is from co-registering the BOLD reference to the same-subject T1w-reference with its intensity inverted [9, 10]. Registration was performed with antsRegistration, and the process was regularized by constraining deformation to be nonzero only along the phase-encoding direction, and modulated with an average fieldmap template [11]. Based on the estimated susceptibility distortion, a corrected EPI (echo-planar imaging) reference was calculated for a more accurate co-registration with the anatomical reference. The BOLD reference was then co-registered to the T1w reference using bbregister (FreeSurfer) that implements boundary-based registration with six DOF [12]. The BOLD time-series were resampled onto native space by applying a single composite transform to correct for head-motion and susceptibility distortions. The BOLD time-series were resampled into MNI152NLin6Asym standard space. Several confounding time-series were calculated based on the preprocessed BOLD including CSF and WM, and the whole-brain masks. The confound time series derived from head motion estimates and global signals were expanded with the inclusion of temporal derivatives and quadratic terms for each [13]. All resamplings were performed with a single interpolation step. Gridded (volumetric) resamplings were performed using antsApplyTransforms configured with Lanczos interpolation to minimize the smoothing effects of other kernels [14]. Non-gridded (surface) resamplings were performed using mri_vol2surf (FreeSurfer).

Modeling Framework

We used a novel mixed-modeling framework to simultaneously study changes in the DMN and across all other regions of the brain [15]. This model is an extension of the model presented in [16] for analyzing whole-brain networks. This new extension enables analyzing local subnetworks within the context of the whole brain network while maintaining the capabilities of the original model. The model is briefly described below.

Let R_{ijk} denote a binary variable which is one if the correlation value between node j and node k of the ith subject's network is positive, and zero otherwise. This variable specifies whether a connection exists between node j and node k of the ith subject's network. Also, let Y_{ijk} denote a continuous variable for positive correlation values (present connections) between node j and node k of the ith subject's network. Then, we can define the following conditional probabilities:

$$P\left(R_{ijk} = 1 | \beta_r; b_{ri}\right) = p_{ijk}(\beta_r; b_{ri}) \quad \textbf{S1}$$

$$P\left(R_{ijk} = 0 | \beta_r; b_{ri}\right) = 1 - p_{ijk}(\beta_r; b_{ri}) \quad \textbf{S2}$$

Where p_{ijk} is the probability of having a connection between node j and node k of the ith subject's network, β_r is the fixed effects (population) parameters vector, and b_{ri} is the random-effects parameter vector modeling correlation (dependence) between repeated network features for subject i. If S_{ijk} denotes a variable specifying present connections between node j and node k of the ith subject's network (i.e., $S_{ijk} = [Y_{ijk}|R_{ijk} = 1]$), we can define the following two-part mixed modeling framework for the probability and strength of brain connections:

$$logit\left(p_{ijk}(\beta_r; b_{ri})\right) = T'_{ijk}\beta_r + Z'_{ijk}b_{ri} \quad \textbf{S3}$$

$$FZT\left(S_{ijk}(\beta_s; b_{si})\right) = T'_{ijk}\beta_s + Z'_{ijk}b_{si} + e_{ijk} \quad \textbf{S4}$$

Where T_{ijk} and Z_{ijk} are design matrices for the fixed- and random-effects, respectively, e_{ijk} captures the random noise (not captured by random effects) in the connection strength between node j and node k of the ith subject's network, and β_s and b_{si} are analogous parameters to β_r and b_{ri}, respectively, but for the connection strength. Equation S3 is a logistic regression model that quantifies the relationship between the connection probability and sets of desired covariates. FZT is the Fisher's Z-transform applied to ensure that normality assumption is met. By including network metrics (such as clustering coefficient and global efficiency) and regional covariates modeling brain subnetworks (DMN in this study), this framework allows testing hypotheses about the DMN and whole brain connectivity and network features, and assessing how covariates of interest affect the connectivity and topology at the local and global levels. For more detail, see the referenced paper. We used the WFU_MMNET toolbox [17] and in-house Matlab scripts to generate the appropriate datasets for this modeling framework, and used SAS v.9.4 to fit the statistical models.

Representative group network generation

Due to the non-Euclidean nature of complex brain networks, it is not possible to generate a group mean network without affecting the overall network topology [18]. In other words, simply averaging connectivity matrices across study participants results in a single group network with topological properties that are not representative of the typical participant [19]. To generate a meaningful visualizations of the significant results from the current study, representative group networks were estimated with respect to the observed modification effects of the covariate of interest on the network variable – connectivity associations. Group networks were estimated from the fitted statistical model in our secondary analysis. The COIs were binary variables separating HIV-MJ-, HIV+MJ-, HIV-MJ+, and HIV+MJ+ (control group). Representative group networks were estimated for the group of subjects with COI = 38 and COI = 60 GlobalT values, within each group. Using average network variables for each group and the estimated parameters along with their standard deviation, the strength of each edge was simulated 20 times (from a normal distribution using estimated parameters and their standard errors), and this whole process was repeated 20 further times and averaged to generate each final edge strength. We only used covariates from the strength model that contributed to the observed modification effect of the COI for each analysis in estimating the representative group networks. All estimated networks were only used to illustrate the significant findings from the statistical analysis. *The simulated group networks were not used to make inferences related to the hypotheses being tested.*

Post-hoc Analyses and Contrast Statements

To test group differences or modification effects in the DMN, further post-hoc analyses were required. Specifically, we estimated the appropriate contrast statements of already estimated residuals for corresponding parameters, to obtain inference about if/how the relationship between network metrics and brain connectivity within the DMN is different between the reference group (HIV-MJ-) and the other three groups (primary analysis), and then to obtain inference about if/how the relationship between the interaction of GlobalT and network metrics and brain connectivity within the DMN was different between the reference group (HIV-MJ-) and the other three groups (secondary analysis). Below, we first briefly explain what each interaction means for HIV versus NonHIV patients (this could be generalized to MJ vs NonMJ as well) and then the interaction estimates used in our contrast statements, along with a detailed description of contrast statements that allowed breaking this even more and comparing HIV+MJ-, HIV-MJ+, and HIV+MJ+, to the reference group are provided.

Summary of Important Interaction Covariates and Contrast Statements for HIV. Figure S1 has been adapted from [3] made available under a Creative Commons Attribution 4.0 International License and is only for illustrative purposes in describing the interaction covariates. Table S1 describes the important interaction covariates.

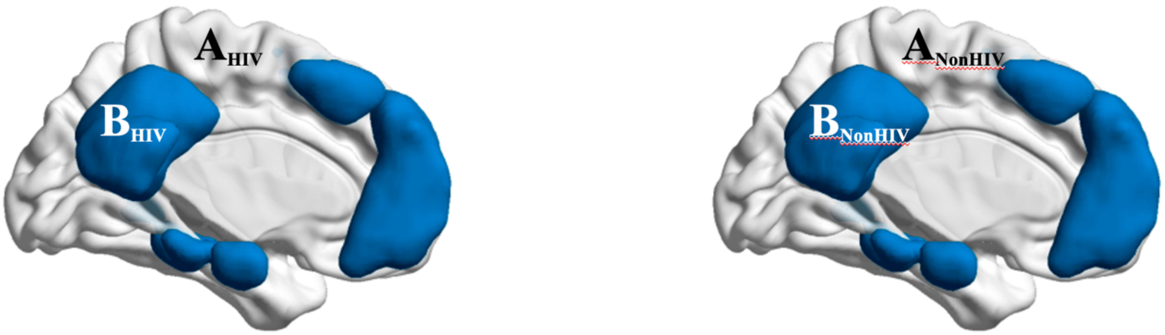

Figure S1. Global and Local (DMN) Regions. The brain networks shown here are identical between the two brains with only different labels for simplicity in describing the interaction covariates. A_{HIV}, A_{NonHIV} show the brain regions excluding the DMN in individuals with (left) and without (right) HIV, respectively – i.e., all regions shown by the white color in both figures. A_{HIV}, A_{NonHIV} show the exact same regions in both groups. B_{HIV}, B_{NonHIV} show the regions within the DMN in individuals with (left) and without (right) HIV, respectively – i.e., regions shown by the blue color in both figures. B_{HIV}, B_{NonHIV} show the exact same regions in both groups.

Table S1. Description of interaction parameters with respect to the regions shown in Fig S2.

$\beta_{HIV\times Clust}$	Is the relationship between connection strength and clustering coefficient different in A_{HIV} and A_{NonHIV}. $(\boldsymbol{\beta_{HIV\times Clust} \sim A_{HIV} - A_{NonHIV}})$
$\beta_{HIV\times Eglobe}$	Is the relationship between connection strength and global efficiency different in A_{HIV} and A_{NonHIV}. $(\boldsymbol{\beta_{HIV\times Eglob} \sim A_{HIV} - A_{NonHIV}})$
$\beta_{HIV\times DMN\times Clust}$	Is the relationship between connection strength and clustering coefficient in B_{HIV} when compared to the same relationship in A_{FW} [i.e., the relationship in ($B_{HIV} - A_{HIV}$)] different than the relationship between connection strength and clustering coefficient in B_{NonHIV} when compared to the same relationship in A_{NonHIV} [i.e., the relationship in (B_{NonHIV} - A_{NonHIV})]. $(\boldsymbol{\beta_{HIV\times DMN\times Clust} \sim [(B_{HIV} - A_{HIV}) - (B_{NonHIV} - A_{NonHIV})]})$
$B_{HIV\times DMN\times Eglobe}$	Is the relationship between connection strength and global efficiency in B_{HIV} when compared to the same relationship in A_{HIV} [i.e., the relationship in (B_{HIV} - A_{HIV})] different than the relationship between connection strength and global efficiency in B_{NonHIV} when compared to the same relationship in A_{NonHIV} [i.e., the relationship in (B_{NonHIV} - A_{NonHIV})]. $(\boldsymbol{\beta_{HIV\times DMN\times Eglob} \sim [(B_{HIV} - A_{HIV}) - (B_{NonHIV} - A_{NonHIV})]})$

According to this table:

$(A_{HIV} - A_{NonHIV}) \sim \beta_{HIV\times Clust}, \beta_{HIV\times Eglobe}$
$[(B_{HIV} - A_{HIV}) - (B_{NonHIV} - A_{NonHIV})] \sim \beta_{HIV\times DMN\times Clust}, \beta_{HIV\times DMN\times Eglobe}$
To find the statistical inference for the group difference ***within*** the DMN – i.e., $(B_{HIV} - B_{NonHIV})$, we will have:
$(B_{HIV} - B_{NonHIV}) = ((A_{HIV} - A_{NonHIV}) + [(B_{HIV} - A_{HIV}) - (B_{NonHIV} - A_{NonHIV})])$
Thus, $(B_{HIV} - B_{NonHIV}) \sim \beta_{HIV\times Clust}, \beta_{HIV\times Eglobe}, \beta_{HIV\times DMN\times Clust}, \beta_{HIV\times DMN\times Eglobe}$

The estimates and p-values for the interaction parameters including $\beta_{HIV\times Clust}, \beta_{HIV\times Eglobe}, \beta_{HIV\times DMN\times Clust}, \beta_{HIV\times DMN\times Eglobe}$ are directly obtained from our statistical model. Using these estimates, we can use make the following contrast statements to test if the DMN organization would be significantly different when comparing the HIV vs NonHIV individuals.
Contrast statement to test the group difference in the DMN clustering between HIV and NonHIV: $(\beta_{COI\times Clust} + \beta_{COI\times DMN\times Clust})$
Contrast statement to test the group difference in the DMN efficiency between HIV and NonHIV: $(\beta_{COI\times Eglobe} + \beta_{COI\times DMN\times Eglobe})$

Note that to test more specific hypotheses (e.g., comparing HIV+MJ+ versus HIV-MJ-), the appropriate interactions with the MJ should be used in the contrast statements as well. But those modifications could be simply applied by using the same process detailed above. Thus, we have not provided the details of making those contrast statements; however, the

final contrast statements used in all reported results for our primary and secondary analyses (Tables 3 and 4 of the paper) are provided in the following.
Key results from our primary analyses (Model 1) that examined the group difference in the clustering and efficiency of the DMN and all other brain regions (as a whole) are presented in **Table 3**. Parameter estimates and (corrected) p-values in this table quantify the group difference between the HIV-MJ- group as our control group and the other three groups. This table shows if/how the relationship of connection strength with network metrics (clustering/efficiency) is different between the control group (HIV-MJ-) – as the reference group in our analyses, and the other three groups, within and outside of the DMN.

Primary Analysis. The contrast statements used in Table 3 (i.e. the contrast statements that allowed quantifying the group difference between the HIV-MJ- group as our control (reference) group and the three other groups including HIV+MJ-, HIV-MJ+, and HIV+MJ+) are provided in Tables S2 and S3 below (SubNet is the DMN covariate):

Tablee S2. contrast statements used in Table 3 for clustering.

Hypothesis	Contrast Statement
HIV+MJ- / Within DMN	Clustering_Coefficient*HIV 1 Clustering_Coefficient*SubNet*HIV 1
HIV-MJ+ / Within DMN	Clustering_Coefficient*MJ 1 Clustering_Coefficient*SubNet*MJ 1
HIV+MJ+ / Within DMN	Clustering_Coefficient*HIV 1 Clustering_Coefficient*SubNet*HIV 1 Clustering_Coefficient*MJ 1 Clustering_Coefficient*SubNet*MJ 1 Clustering_Coefficient*HIV*MJ 1 Clustering_Coefficient*SubNet*HIV*MJ 1
HIV+MJ- / Outside DMN	Clustering_Coefficient*HIV 1 Clustering_Coefficient*SubNet*HIV 0
HIV-MJ+ / Outside DMN	Clustering_Coefficient*MJ 1 Clustering_Coefficient*SubNet*MJ 0
HIV+MJ+ / Outside DMN	Clustering_Coefficient*HIV 1 Clustering_Coefficient*MJ 1 Clustering_Coefficient*HIV*MJ 1 Clustering_Coefficient*SubNet*HIV 0 Clustering_Coefficient*SubNet*MJ 0

The contrast statements in this table and Table S3 were used in SAS GLIMMIX.

Tablee S3. contrast statements used in Table 3 for efficiency.

Hypothesis	Contrast Statement
HIV+MJ- / Within DMN	Global_Efficiency*HIV 1 Global_Efficiency *SubNet*HIV 1
HIV-MJ+ / Within DMN	Global_Efficiency *MJ 1 Global_Efficiency *SubNet*MJ 1
HIV+MJ+ / Within DMN	Global_Efficiency *HIV 1 Global_Efficiency *SubNet*HIV 1 Global_Efficiency *MJ 1 Global_Efficiency *SubNet*MJ 1 Global_Efficiency *HIV*MJ 1 Global_Efficiency *SubNet*HIV*MJ 1
HIV+MJ- / Outside DMN	Global_Efficiency *HIV 1 Global_Efficiency *SubNet*HIV 0
HIV-MJ+ / Outside DMN	Global_Efficiency *MJ 1 Global_Efficiency *SubNet*MJ 0
HIV+MJ+ / Outside DMN	Global_Efficiency *HIV 1 Global_Efficiency *MJ 1 Global_Efficiency *HIV*MJ 1 Global_Efficiency *SubNet*HIV 0 Global_Efficiency *SubNet*MJ 0

The estimates, standard errors, and the p-values obtained for the interaction covariates that were used for testing the hypotheses presented in Table 3 of the paper (the estimates that were used in our contrast statements (Tables S2 and S3)) are presented in Table S4. These estimates were the direct outputs from our statistical model. The residuals obtained for these estimates were used in our contrast statements described above in Tables S2 and S3.

Tablee S4. Estimates, standard error, and p-values obtained from SAS GLIMMIX

Parameter	Estimate	Standard Error	t Value	Pr > |t|
Clustering_Coeff*HIV	0.003627	0.004956	0.73	0.4643
Clustering_Coef*HIV*MJ	-0.00483	0.007097	-0.68	0.4960
Clustering_Coeffi*MJ	0.003719	0.005157	0.72	0.4708
Clustering_Coef*SubNet	-0.04190	0.001630	-25.71	<.0001
Clustering_Coef*HIV*SubNet	-0.00508	0.002146	-2.37	0.0179
Clustering_Coef*HIV*MJ*SubNet	-0.00452	0.003186	-1.42	0.1557
Clustering_Coef*MJ*SubNet	-0.01534	0.002361	-6.50	<.0001
Global_Efficiency*HIV	-0.00263	0.003469	-0.76	0.4487
Global_Efficiency*HIV*MJ	0.003078	0.004969	0.62	0.5356
Global_Efficiency*MJ	-0.00242	0.003612	-0.67	0.5023
Global_Efficiency*SubNet	0.03957	0.001670	23.69	<.0001
Global_Efficiency*HIV*SubNet	0.01460	0.002231	6.54	<.0001
Global_Efficiency*HIV*MJ*SubNet	-0.00285	0.003290	-0.87	0.3858
Global_Efficiency*MJ*SubNet	0.02405	0.002438	9.86	<.0001

The contrast statements used in fining the line plot slopes in Fig 1 (only for visualization and helping with the interpretation of the results in Table 3) are presented in Table S5 and S6.

Tablee S5. contrast statements used in Fig 1.A

Group	Contrast Statement
HIV-MJ-	Clustering_Coefficient 1 Clustering_Coefficient*SubNet 1
HIV+MJ-	Clustering_Coefficient 1 Clustering_Coefficient*SubNet 1 Clustering_Coefficient*HIV 1 Clustering_Coefficient*SubNet*HIV 1
HIV-MJ+	Clustering_Coefficient 1 Clustering_Coefficient*SubNet 1 Clustering_Coefficient*MJ 1 Clustering_Coefficient*SubNet*MJ 1
HIV+MJ+	Clustering_Coefficient 1 Clustering_Coefficient*SubNet 1 Clustering_Coefficient*HIV 1 Clustering_Coefficient*SubNet*HIV 1 Clustering_Coefficient*MJ 1 Clustering_Coefficient*SubNet*MJ 1 Clustering_Coefficient*HIV*MJ 1 Clustering_Coefficient*SubNet*HIV*MJ 1

Tablee S6. contrast statements used in Fig 1.B

Group	Contrast Statement
HIV-MJ-	Global_Efficiency 1 Global_Efficiency*SubNet 1;
HIV+MJ-	Global_Efficiency 1 Global_Efficiency*SubNet 1 Global_Efficiency*HIV 1 Global_Efficiency*SubNet*HIV 1;
HIV-MJ+	Global_Efficiency 1 Global_Efficiency*SubNet 1 Global_Efficiency*MJ 1 Global_Efficiency*SubNet*MJ 1;
HIV+MJ+	Global_Efficiency 1 Global_Efficiency*SubNet 1 Global_Efficiency*HIV 1 Global_Efficiency*SubNet*HIV 1 Global_Efficiency*MJ 1 Global_Efficiency*SubNet*MJ 1 Global_Efficiency*HIV*MJ 1 Global_Efficiency*SubNet*HIV*MJ 1;

Secondary Analysis. The contrast statements used in Table 4 (i.e. the contrast statements that allowed quantifying the group difference in the association of DMN organization and GlobalT) are presented in Tables S7 and S8 below.

Tablee S7. contrast statements used in Table 4 for clustering.

Hypothesis	Contrast Statement
HIV+MJ- / Within DMN	Clustering_Coefficient*COI*HIV 1 Clustering_Coefficient*COI*SubNet*HIV 1
HIV-MJ+ / Within DMN	Clustering_Coefficient*COI*MJ 1 Clustering_Coefficient*COI*SubNet*MJ 1
HIV+MJ+ / Within DMN	Clustering_Coefficient*COI*HIV 1 Clustering_Coefficient*COI*SubNet*HIV 1 Clustering_Coefficient*COI*MJ 1 Clustering_Coefficient*COI*SubNet*MJ 1 Clustering_Coefficient*COI*HIV*MJ 1 Clustering_Coefficient*COI*SubNet*HIV*MJ 1
HIV+MJ- / Outside DMN	Clustering_Coefficient*COI*HIV 1 Clustering_Coefficient*COI*SubNet*HIV 0
HIV-MJ+ / Outside DMN	Clustering_Coefficient*COI*MJ 1 Clustering_Coefficient*COI*SubNet*MJ 0
HIV+MJ+ / Outside DMN	Clustering_Coefficient*COI*HIV 1 Clustering_Coefficient*COI*MJ 1 Clustering_Coefficient*COI*HIV*MJ 1 Clustering_Coefficient*COI*SubNet*HIV 0 Clustering_Coefficient*COI*SubNet*MJ 0

The contrast statements in this table and Table S8 were used in SAS GLIMMIX.

Tablee S8. contrast statements used in Table 4 for efficiency.

Hypothesis	Contrast Statement
HIV+MJ- / Within DMN	Global_Efficiency*COI*HIV 1 Global_Efficiency*COI*SubNet*HIV 1
HIV-MJ+ / Within DMN	Global_Efficiency*COI*MJ 1 Global_Efficiency*COI*SubNet*MJ 1
HIV+MJ+ / Within DMN	Global_Efficiency*COI*HIV 1 Global_Efficiency*COI*SubNet*HIV 1<br>Global_Efficiency*COI*MJ 1 Global_Efficiency*COI*SubNet*MJ 1<br>Global_Efficiency*COI*HIV*MJ 1 Global_Efficiency*COI*SubNet*HIV*MJ 1
HIV+MJ- / Outside DMN	Global_Efficiency*COI*HIV 1 Global_Efficiency*COI*SubNet*HIV 0
HIV-MJ+ / Outside DMN	Global_Efficiency*COI*MJ 1 Global_Efficiency*COI*SubNet*MJ 0
HIV+MJ+ / Outside DMN	Global_Efficiency*COI*HIV 1 Global_Efficiency*COI*MJ 1<br>Global_Efficiency*COI*HIV*MJ 1 Global_Efficiency*COI*SubNet*HIV 0<br>Global_Efficiency*COI*SubNet*MJ 0

The estimates, standard errors, and the p-values obtained for the interaction covariates that were used for testing the hypotheses presented in Table 4 of the paper (the estimates that were used in our contrast statements (Tables S7 and S8)) are presented in Table S9. These estimates were the direct outputs from our statistical model. (Note that all two-way, three-way, and four-way interactions that were used in the model but not in the contrast statement are not shown in this table for simplicity). The residuals obtained for these estimates were used in our contrast statements described above in Tables S7 and S8.

Tablee S9. Estimates, standard error, and p-values obtained from SAS GLIMMIX

Parameter	Estimate	Standard Error	t Value	Pr > |t|
Clustering_Coef*COI*HIV	-0.00638	0.005048	-1.26	0.2066
Clustering_Coef*COI*HIV*MJ	0.008321	0.007320	1.14	0.2556
Clustering_Coef*COI*MJ	0.001120	0.005076	0.22	0.8254
Clustering_Coef*COI*HIV*SubNet	0.02911	0.002549	11.42	<.0001
Clustering_Coef*COI*HIV*MJ*SubNet	-0.02770	0.003599	-7.70	<.0001
Clustering_Coef*COI*MJ*SubNet	0.01627	0.002300	7.08	<.0001
Global_Efficiency*COI*HIV	0.004277	0.003566	1.20	0.2304
Global_Efficiency*COI*HIV*MJ	-0.00670	0.005173	-1.29	0.1954
Global_Efficiency*COI*MJ	-0.00055	0.003587	-0.15	0.8776
Global_Efficiency*COI*HIV*SubNet	-0.02506	0.002420	-10.35	<.0001
Global_Efficiency*COI*HIV*MJ*SubNet	0.02210	0.003501	6.31	<.0001
Global_Efficiency*COI*MJ*SubNet	-0.01548	0.002330	-6.64	<.0001

COI represents GlobalT in this table.

The estimates, standard errors, and the p-values obtained for the confounding covariates form our secondary anaysis are presented in Table S10. As this table shows, age, gender, and average_RMD, are significantly associated with the connection strength outside of the DMN. Future studies will examine this in more detail as we only used these variables as confounding covariates in our analyses.

Tablee S10. Estimates, standard error, and p-values obtained from SAS GLIMMIX

Parameter	Estimate	Standard Error	t Value	Pr > |t|
Age	0.006916	0.003060	2.26	0.0238
Gender	-0.03371	0.007460	-4.52	<.0001
Race	0.009032	0.006434	1.40	0.1604
Education	-0.00048	0.003193	-0.15	0.8812
Average_RMD	-0.00907	0.003042	-2.98	0.0029

Table S11. P-values for comparing the groups using two-tailed t-test

	Control /HIV+MJ-	Control /HIV-MJ+	Control /HIV+MJ+	HIV+MJ- /HIV-MJ+	HIV+MJ- /HIV+MJ+	HIV-MJ+/ HIV+MJ+
**GlobalT**	0.1196	0.8975	**0.0020**	0.0915	0.0945	**0.0013**
**Age**	0.8830	0.1381	0.5166	0.0768	0.3929	0.3763
**Sex**	---	---	---	---	---	---
**Education**	**0.0438**	**< 0.0001**	**< 0.0001**	**0.0323**	**0.0127**	0.7932
**Race**	---	---	---	---	---	---
**Average RMD**	0.8694	0.9054	0.5580	0.9643	0.7120	0.6762

Bold values show the Significant differences

Table S12. Average network density across the four studied groups (mean±std)

	HIV-MJ-	HIV-MJ+	HIV+MJ-	HIV+MJ+
Network Density	**0.6698 ± 0.0737**	**0.6691 ± 0.0742**	**0.6678 ± 0.0773**	**0.6752 ± 0.0670**

Table S13. Pairwise group comparisons of network density (p-values)

	HIV-MJ- / HIV-MJ+	HIV-MJ- / HIV+MJ-	HIV-MJ- / HIV+MJ+	HIV-MJ+ / HIV+MJ-	HIV-MJ+ / HIV+MJ+	HIV+MJ- / HIV+MJ+
p-values	**0.9640**	**0.8877**	**0.6718**	**0.9265**	**0.6459**	**0.5707**

Table S14. Correlation of network metrics with interaction terms

Interaction Covariates	Clustering Coefficient	Interaction Covariates	Global Efficiency
Clustering*HIV	-0.7025	Efficiency*HIV	-0.6944
ClusteringHIV*MJ	0.4906	Efficiency*HIV*MJ	0.4849
Clustering*MJ	-0.6751	Efficiency*MJ	-0.6671
Clustering*SubNet	-0.02177	Efficiency*SubNet	-0.02750
Clustering*HIV*SubNet	0.01665	Efficiency*HIV*SubNet	0.02071
Clustering*HIV*MJ*SubNet	-0.01123	Efficiency*HIV*MJ*SubNet	-0.01407
Clustering*MJ*SubNet	0.01516	Efficiency*MJ*SubNet	0.01898

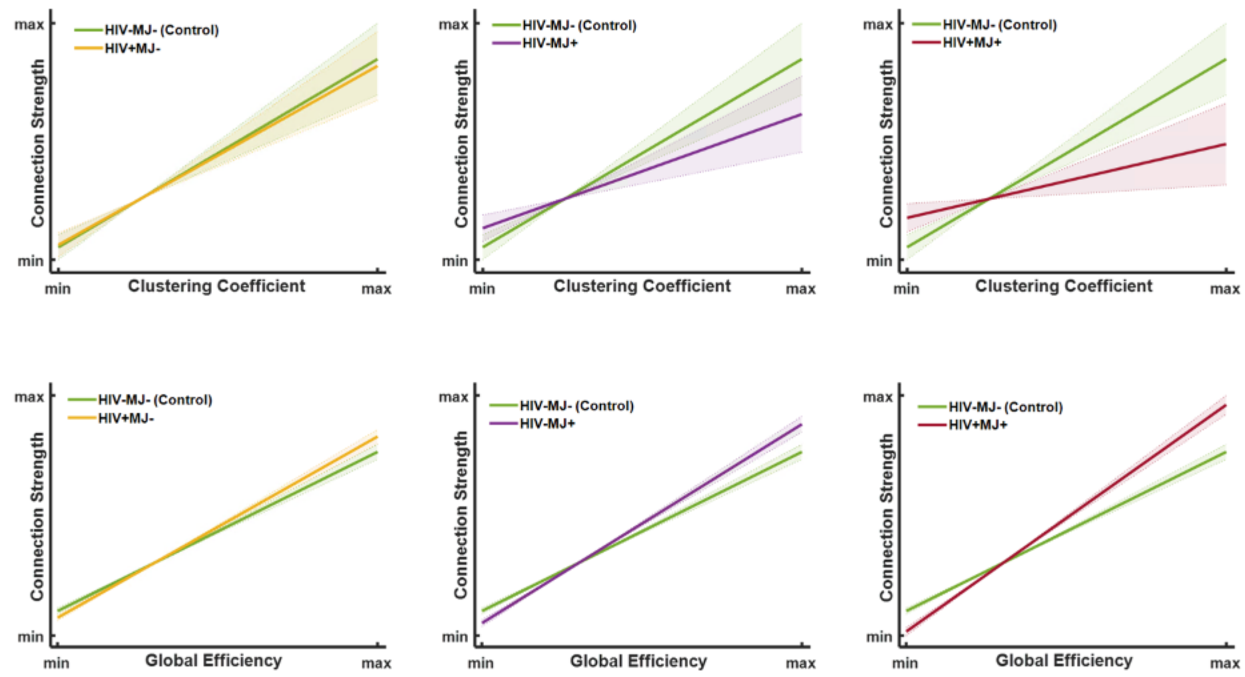

Figure S2. Line plots with 95% confidence intervals demonstrating the relationship of DMN connection strength with clustering (A) and efficiency (B) for all groups.

References

1. Tustison, N.J., et al., *N4ITK: improved N3 bias correction.* IEEE transactions on medical imaging, 2010. **29**(6): p. 1310.
2. Avants, B.B., et al., *Symmetric diffeomorphic image registration with cross-correlation: Evaluating automated labeling of elderly and neurodegenerative brain.* Medical Image Analysis, 2008. **12**(1): p. 26-41.
3. Zhang, Y., M. Brady, and S. Smith, *Segmentation of brain MR images through a hidden Markov random field model and the expectation-maximization algorithm.* IEEE Transactions on Medical Imaging, 2001. **20**(1): p. 45-57.
4. Dale, A.M., B. Fischl, and M.I. Sereno, *Cortical surface-based analysis - I. Segmentation and surface reconstruction.* Neuroimage, 1999. **9**(2): p. 179-194.
5. Klein, A., et al., *Mindboggling morphometry of human brains.* Plos Computational Biology, 2017. **13**(2): p. e1005350.
6. Evans, A.C., et al., *Brain templates and atlases.* NeuroImage, 2012. **62**(2): p. 911-22.
7. Jenkinson, M., et al., *Improved optimization for the robust and accurate linear registration and motion correction of brain images.* NeuroImage, 2002. **17**(2): p. 825-41.
8. Cox, R.W. and J.S. Hyde, *Software tools for analysis and visualization of fMRI data.* NMR Biomed, 1997. **10**(4-5): p. 171-8.
9. Wang, S., et al., *Evaluation of Field Map and Nonlinear Registration Methods for Correction of Susceptibility Artifacts in Diffusion MRI.* Front Neuroinform, 2017. **11**: p. 17.
10. Huntenburg, J.M. *Evaluating nonlinear coregistration of BOLD EPI and T1w images*. 2014; Available from: http://hdl.handle.net/11858/00-001M-0000-002B-1CB5-A.
11. Treiber, J.M., et al., *Characterization and Correction of Geometric Distortions in 814 Diffusion Weighted Images.* PLoS One, 2016. **11**(3): p. e0152472.
12. Greve, D.N. and B. Fischl, *Accurate and robust brain image alignment using boundary-based registration.* Neuroimage, 2009. **48**(1): p. 63-72.
13. Satterthwaite, T.D., et al., *An improved framework for confound regression and filtering for control of motion artifact in the preprocessing of resting-state functional connectivity data.* NeuroImage, 2013. **64**: p. 240-256.
14. Lanczos, C., *Evaluation of Noisy Data.* Journal of the Society for Industrial and Applied Mathematics Series B Numerical Analysis, 1964. **1**(1): p. 76-85.
15. Bahrami, M., P.J. Laurienti, and S.L. Simpson, *Analysis of brain subnetworks within the context of their whole-brain networks.* Human Brain Mapping, 2019. **40**(17): p. 5123-5141.
16. Simpson, S.L. and P.J. Laurienti, *A two-part mixed-effects modeling framework for analyzing whole-brain network data.* Neuroimage, 2015. **113**: p. 310-319.
17. Bahrami, M., P.J. Laurienti, and S.L. Simpson, *A MATLAB toolbox for multivariate analysis of brain networks.* Human Brain Mapping, 2019. **40**(1): p. 175-186.
18. Hayasaka, S. and P.J. Laurienti, *Comparison of characteristics between region-and voxel-based network analyses in resting-state fMRI data.* Neuroimage, 2010. **50**(2): p. 499-508.
19. Simpson, S.L., M.N. Moussa, and P.J. Laurienti, *An exponential random graph modeling approach to creating group-based representative whole-brain connectivity networks (vol 60, pg 1117, 2012).* Neuroimage, 2012. **62**(3): p. 2178-2178.
